# Determinants of Delay in Seeking Malaria Treatment for Under-Five Children at Gambella Town, Southwest Ethiopia: A Case-Control Study

**DOI:** 10.1155/2020/2310971

**Published:** 2020-08-10

**Authors:** Addisu Tona Shumerga, Habtamu Jarso Hebo, Tsegaye Tewelde Gebrehiwot, Mamo Nigatu Gebre

**Affiliations:** ^1^Gambella People's National Regional State Health Bureau, Gambella, Ethiopia; ^2^Department of Epidemiology, Faculty of Public Health, Institute of Health, Jimma University, Jimma, Ethiopia

## Abstract

**Background:**

Most of the malaria-related complications in children are due to delay in the treatment-seeking of caregivers. The objective of this study was to identify determinants of delay in seeking malaria treatment for under-five children in Gambella town, Ethiopia.

**Methods:**

A case-control study was conducted in March 2017 among caregivers/parents consecutively included in the study. Data were collected by face-to-face interviews using a structured questionnaire. Descriptive statistics and logistic regression were, respectively, used for descriptive and analytical data analyses. Adjusted odds ratios and 95% CI were, respectively, calculated to assess the strength of association and statistical significance.

**Result:**

A total of 153 cases and 153 control caregivers/parents participated in the study giving a response rate of 100%. The mean age of cases and controls was 29.4 years (SD ± 6.0 years) and 29.63 years (SD ± 7.8 years), respectively. Being housewife (AOR = 2.50; 95% CI: 1.47–4.22), having no history of child mortality (AOR = 3.70; 95% CI: 1.79–7.64), and chewing khat (AOR = 3.50; 95% CI: 1.57–7.68) were significantly associated with delay in seeking malaria treatment for under-five children among the caregivers. *Conclusion and Recommendation.* Comprehensive community-based malaria prevention and control education should be given for the caregivers in the town giving due emphasis to housewives, khat-chewers, and the caregivers with no story of child death to better promote early malaria treatment-seeking for under-five children.

## 1. Introduction

Malaria is a life-threatening disease caused by *Plasmodium* parasites that are transmitted to people through the bites of infected female *Anopheles* mosquitoes, called malaria vector [1–3]. The four most common human malaria parasites are *Plasmodium falciparum*, *Plasmodium vivax*, *Plasmodium ovale*, and *Plasmodium malariae*, of which *Plasmodium falciparum* is most common across sub-Saharan Africa [1]. In Ethiopia, *plasmodium falciparum* and *Plasmodium vivax* are the most dominant malaria parasites distributed all over the country and accounting for 60% and 40% of malaria cases, respectively, whereas *Plasmodium malariae* accounts for less than 1%, and *Plasmodium ovale* is rarely reported [4, 5]. The disease is characterized by symptoms such as fever, headache, backache, joint pains, and vomiting. If it is not treated early, even the uncomplicated case can progress rapidly to severe forms of the disease and eventually may result in death [1, 3]. Malaria occurrence mainly depends on climatic factors such as temperature, humidity, and rainfall. Malaria is mainly transmitted in tropical and subtropical areas where *Anopheles* mosquitoes can survive and multiply and the malaria parasites can complete their growth cycle in the mosquitoes [1, 6].

Regardless of collective efforts put in a place to prevent and control malaria, the disease has continued to be the major public health problem. According to the 2020 WHO and CDC report, globally, there were an estimated 228 million cases of malaria in 2018, whereas 405,000 people died from the disease in the same year [3, 7]. The WHO African Region disproportionately carries a high share of the global burden of the disease accounting for 93% of cases and 94% of deaths in 2018 [3, 7, 8]. Malaria is one of the leading top ten causes of death in Ethiopia where its transmission pattern and intensity are different due to variations in altitude, rainfall, and population movement [4, 5]. According to the 2019 U.S President's Malaria Initiative Malaria Operational Plan report, there were 1,755,748 total cases of malaria in 2017, whereas 60 million people were at risk of contracting the disease in Ethiopia [4]. Even though malaria morbidity and mortality have significantly decreased in Ethiopia since 2000, its transmission remains high in the western border area near Sudan and South Sudan [9]. Children under five years of age are the most vulnerable group affected by malaria that accounted for 67% of all malaria deaths worldwide in 2018 [3].

While prompt diagnosis and treatment is the most effective way to prevent a mild case of malaria from developing into severe disease and death [8, 10], based on the secondary data analyses of household surveys completed in 20 sub-Saharan Africa countries between 2015 and 2018, a high proportion (36%) of febrile children did not receive any medical attention [8]. Studies conducted in the southwest and southern part of Ethiopia identified that complaining about the side effects of antimalarial drugs, distance form health facility, and cost of transportation to reach the health facilities were factors that delayed malaria treatment-seeking. In addition to this, monogamous marriage, khat chewing, sex of the child, and absence history of child death in the family were factors counted for the delay in treatment-seeking for malaria [11, 12].

According to the analysis of the data from different health facilities of the Gambella region, most of the malaria-related complications among the children of the region were due to delay in treatment-seeking. Despite this, as far as the investigators' knowledge, there is no previous study depicting the causes of this delayed treatment-seeking among caregivers of under-five children in the region. Therefore, the current study is aimed to identify predictors of delay in malaria treatment-seeking, which will be very crucial to set strategies to solve the problem in the study area and similar settings.

## 2. Methods

### 2.1. Study Area

The study was conducted in under-five outpatient department of Gambella Hospital and Gambella town health center, Gambella town. Gambella town is a separate district, and the capital of the Gambella region is located at the confluence of the Baro River and its tributary the Jajjaba. The town has a latitude and longitude of 8°15′N 34°35′E and has an elevation of 526 meters above sea level having a hot climatic condition with an annual average temperature range from 20°C to 28°C. Gambella town is located 768 kilometers in the southwest of Ethiopia away from Addis Ababa.

The town harbors different ethnic groups. The majority of ethnic groups residing in the town are Nuer, Agnuha, and Mejanger. However, there are also other ethnic groups including settlers from other parts of the country. The total projected population size of Gambella town in 2017 was 74,102, of whom 52.8% are men [13]. The town had a total of 16,109 households with an average of 4.6 persons in a household. The town has one hospital, one health center, one governmental junior clinic, and 18 private clinics. The two governmental health facilities in the town (Gambella Hospital and Gambella town health center) were included in the study as the majority of the town population is treated there. The livelihood of the population is mainly dependent on government work and trade.

### 2.2. Study Design and Population

The study was conducted from March 1 to 26, 2017. A facility-based case-control study design was used. The study was conducted among caregivers who took treatment from government health facilities alone due to the reason that most of the private clinics had no regular full-time working health professionals and adequate laboratory facilities for malaria treatment. The cases were caregivers/parents of under-five children with malaria presented at a health facility to seek treatment after 24 hours from the first time of observing signs and symptoms presumptuous of malaria, whereas controls were caregivers/parents of under-five children with malaria who came to a health facility to seek treatment within 24 hours from the first time of observing signs and symptoms presumptuous of malaria. Caregivers who sought treatment from other health facilities in the town before they came to the study areas and caregivers whose age was less than 15 years were excluded from this study. We excluded caregivers aged less than 15 years of age for ethical issues.

### 2.3. Sample Size and Sampling Technique

The sample size was calculated using two population proportion formula for case-control study design based on the following assumptions: 1:1 control to case ratio, 2.6% fear of drug side effect in the controls [11], 4.96 odds ratio, 80% power, 95% confidence level, and 5% nonresponse rate. The final sample size was 306 caregivers of under-five children (153 cases and 153 controls).

The proportional allocation of samples to each health facility was performed based on cases obtained in the first week during the data collection period. Accordingly, 220 (110 cases and 110 controls) and 86 (43 cases and 43 controls) were selected by nonduplicative consecutive sampling techniques from Gambella Hospital and Gambella town health center, respectively. When a caregiver came to a health facility with more than one malaria-infected children at a time, only one child was randomly included.

### 2.4. Data Collection

Variables included in the study were sociodemographic/economic characteristics of caregivers/parents which comprise sex, age, religion, ethnic group, education status, marital status, monthly income, and occupational status; child characteristics which comprise age and sex of the child; household factors which comprise the number of under-five children in the household, family size and biological parents living in the home; transportation factors which comprise distance from health facility and type of transportation system; and behavioral factors which comprise alcohol drinking, khat chewing, fear of drug side effect, history of child death, distrust in health providers, knowledge about malaria self-medication, visiting traditional healers, and fear of treatment cost. Prior to the main study, pretesting of the tool was conducted in Itang woreda (i.e., Itang health center) on 15 caregivers of under-five children.

Under-five children presented at the under-five outpatient department were diagnosed for malaria by a health personnel who had been working in the department. Children suspected of malaria by a health personnel working in the under-five outpatient department were sent to the laboratory for confirmation of malaria using an electrical microscope. After the diagnosis, eligible caregivers of malaria-infected under-five children were recruited for the study. An exit face-to-face interviews were conducted to collect data using a structured questionnaire adapted from different related literature [11, 14–16]. Data collectors were four diploma nurses who fluently speak both local language and Amharic who were supervised by two BSc public health officers. As almost all the town residents speak Amharic, Amharic language was used for the data collection. Where caregivers were not able to speak Amharic, the questionnaires were delivered by their native languages as our data collectors were fluent in all languages spoken in the town. The supervisors for data collection checked the questionnaire for completeness and consistency and solved emanating problems during data collection. The reliability of the tool was checked using Cronbach's alpha and yielded a score of 0.732. The data collection tool was refined based on the findings from the pretesting.

### 2.5. Data Processing and Analysis

The collected data were checked for completeness and consistency and coded manually. Data were entered in EpiData version 3.1 and exported to SPSS version 21 for statistical analysis. First univariate analysis was conducted for data exploration and descriptive analysis.

Bivariate analysis was conducted to identify candidate variables associated with a delay in seeking malaria treatment for inclusion in multivariable analysis. Multivariable logistic regression was applied by a backward elimination method. All predictor variables that were associated with the dependent variable in bivariate analysis with a *p* value of less than or equal to 0.25 were included in the model of multivariable analysis. Multicollinearity between significant variables in bivariate analysis with a *p* value of less than 0.25 was checked by a variance inflation factor (VIF) before entering into a multivariable regression. The model fitness was checked by Hosmer–Lemeshow goodness of fit tests, which yield a *p* value of greater than 0.05. Crude and adjusted odds ratios with 95% confidence interval were computed.

### 2.6. Ethical Consideration

An ethical approval letter was obtained from the Institutional Review Board, Institute of Health, Jimma University. Permission to conduct the study was obtained from Gambella Health Bureau, Gambella Hospital, and Gambella town health center. Verbal informed consent was obtained from study participants after providing detailed information on objectives, the possible benefits, and risks posed by the study. The questionnaires were coded anonymously to ensure that the confidentiality of participants was maintained. Besides, the data were collected in a separate room to maintain the privacy of study participants.

### 2.7. Operational Definitions

If caregivers/parents treat their child at home without seeking advice from health providers by using any amount and type of treatment, it was considered as self-medication. On the other hand, if caregivers/parents visited traditional healers to seek treatment at least for a single occasion prior to presenting to the health facility for seeking treatment for their children, it was considered as visiting traditional healers. Distance from health facility was measured by the number of kilometers found between the caregiver's home and the nearest health facility. Knowledge status of the caregivers was assessed by nine questions on the cause, sign/symptoms, preventions, and controls methods of malaria and categorized as good and poor knowledge by using the mean score [11]. Caregivers/parents who had scored equal or more than 50% of questions related to symptoms, causes, transmissions, and prevention mechanisms of malaria were considered as having good knowledge of the disease, whereas caregivers/parents who had scored below 50% of questions related to symptoms, causes, transmissions, and prevention mechanisms of malaria were considered as having poor knowledge of the disease. In the current study, the mother's education, father's education, mother's occupation, and father's occupation refer to the attributes of the children included in the study.

## 3. Results

### 3.1. Sociodemographic/Economic Characteristics

A total of 153 cases and 153 controls were included in this study. Family's monthly income information was missing for 51 (16.67%) caregivers of under-five children; therefore, they were dropped from the analysis of data on monthly income. The mean age of cases and controls was 29.4 years (SD ± 6.0) and 29.6 years (SD ± 7.8), respectively. The majority, 47.7% of cases were Protestants, whereas 39.9% of controls were orthodox Christians. Agnuha was the largest ethnic group for both cases (26.8%) and controls (24.8%). 31.4% of cases could not read and write, whereas 24.8% of controls completed secondary education and above. 31.7% of fathers of under-five children of cases and 64.5% of fathers of under-five children of controls completed secondary education and above. The majority of both cases (64.7%) and controls (41.4%) were housewives. The majority of cases (98.7%) and controls (96.1%) were married (Table 1).

### 3.2. Caregiver, Child, Household, and Accessibility to a Health Facility

The majority of children of both cases (71.9%) and controls (76.5%) live together with both biological mothers and fathers. The majority of cases (81%) and controls (86.3%) lived at a distance of 3 km or less from the health facility. The majority of both cases (75.2%) and controls (77.8%) used public transport to arrive at the facility.

In bivariate logistic regression, sex of caregivers, age of caregivers, religion, ethnic group, mother's education, father's education, mother's occupation, father's occupation, sex of the child, age of the child, family size, and distance from a health facility were candidate variables for multivariable analysis with a *p* value of less than 0.25 (Table 1).

### 3.3. Behavioral Related Factors

The leading percent of cases (90.2%) and controls (77.8%) had no history of child death. The above three-quarters of both cases (81.7%) and controls (86.3%) had good knowledge of the prevention and control of malaria.

In bivariate logistic regression, absence of a history of child death, distrust at health providers, self-medication, and khat chewing were candidate variables for multivariable analysis (Table 2).

### 3.4. Determinants of Delay in Seeking Malaria Treatment

Multivariable logistic regression was fitted to identify factors independently associated with the delay in seeking treatment and to control for confounders. Accordingly, three variables were independently associated with seeking malaria treatment for under-five children in Gambella town.

A housewife caregiver was 2.5 times more likely to delay seeking malaria treatment compared to a caregiver who was employed (AOR = 2.50; 95% CI: 1.47–4.22). A caregiver who had no history of child mortality was nearly four times more likely to delay seeking malaria treatment compared to their counterparts (AOR = 3.70; 95% CI: 1.79–7.64). A caregiver of the child who chews khat was 3.5 times more likely to delay seeking malaria treatment than a caregiver who does not chew khat (AOR = 3.50; 95% CI: 1.57–7.68) (Table 3).

## 4. Discussion

This study identified determinants of delay in seeking malaria treatments in under-five children in Gambella town. Being housewife, absence of a history of child death in the family, and khat chewing were independent predictors of delay in seeking malaria treatment in Gambella town.

A housewife mother was approximately three times more likely to delay seeking malaria treatment for her child/children than a mother who was employed, which is supported by studies conducted in Nigeria where employed mothers were more likely to seek treatment for their children earlier than unemployed mothers [15, 16]. However, this finding was in contrast to the finding of studies conducted in the Bata district, Equatorial Guinea, and the southwest part of Ethiopia, which showed no association between mother's occupation and delay in seeking malaria treatment [11, 17]. This discrepancy might be due to differences in social influence on the importance of early treatment and/or impact of delay on the disease and treatment in the areas. Housewife caregivers in the current study area might have little information on the importance of early treatment because they might have lower contact with those who have good knowledge of it.

This study revealed that having no history of child mortality in the family was independently associated to delay in seeking malaria treatment for under-five children in Gambella town. A caregiver who had no history of child death in the family was approximately four times more likely to delay in seeking malaria treatment compared to a caregiver who had a history of child mortality. This finding is similar to the studies conducted in the southwest part of Ethiopia and Shashogo woreda of southern Ethiopia [11, 12]. This might be due to the reason that caregivers who had the history of child death had taken a lesson from the previous child loss because such trauma might shape the treatment-seeking behavior of caregivers.

Khat chewing was also significantly associated to delay in seeking malaria treatment among caregivers of under-five children. A caregiver who chews khat was nearly four times more likely to delay in seeking malaria treatment for their children compared to a caregiver who does not chew khat, which is in line with a study conducted in Southern Ethiopia [12]. This might be explained by different reasons. Since chewing khat is time-intensive, khat-chewers waste most of their time on chewing, which affects their focuses (close follow-up) to control the health of their children. On the other hand, khat chewing devastatingly affects the financial status of caregivers and delays treatment-seeking for the infected child/children due to the perceived or real cost of treatment.

Chewing khat, as evidenced with different studies [18, 19], was also associated with different health problems such as depression, anxiety, and stress, which might hamper health-seeking behavior. It is also associated with other mental and physical health problems and may interfere with the health-seeking behavior of caregivers [20, 21].

Different studies showed that distance from nearby health facilities was associated with delay [11, 12, 14, 15, 22]. For example, a study conducted in Southwest Ethiopia found that mothers who lived at a distance of more than three kilometers from a health facility were more probably delayed in seeking treatment compared to caregivers who lived within a radius of three kilometers from a health facility [11]. However, the current study showed that there was no association between distance and delay in seeking malaria treatment. This might be due to the reason that the majority of study participants in the current study live within a distance of three kilometers radius from the nearby health facility.

This study has its limitations; in that, the study was conducted on government health facilities only due to the reason that most of the private clinics had no regular full-time working health professionals and adequate laboratory facilities. Therefore, the result of this study cannot be generalized to the population in the town.

## 5. Conclusion and Recommendations

This study revealed that being a housewife, absence of a history of child death in the family, and khat chewing were independently associated with the delay in seeking malaria treatment among caregivers of under-five children in Gambella town. Comprehensive community-based malaria prevention and control education should be given for the caregivers giving due emphasis to housewife, khat-chewers, and caregivers with no story of child death to better promote early malaria treatment-seeking for under-five children among the caregivers.

## Figures and Tables

**Table 1:** Caregiver, child, household, and accessibility to health facility-related factors among caregivers of under-five children in Gambella town, March 2017 (*N* = 306).

Variables	Delay status in malaria treatment		Total (%)	Crude OR (95% CI)	*p* value
	Case (*N* = 153) number (%)	Control (*N* = 153) number (%)			
Sociodemographic/economic characteristics					
Sex					
Female	111 (72.5)	100 (65.4)	211 (69.0)	1.40 (0.86, 2.28)	0.175
Male	42 (27.5)	53 (34.6)	95 (31.0)	1	
Age					
<20	9 (5.9)	14 (9.2)	23 (7.5)	1	
20–29	77 (50.3)	77 (50.3)	154 (50.3)	1.56 (0.64, 3.81)	0.333
30–39	59 (38.6)	42 (27.5)	101 (33.0)	2.18 (0.86, 5.51)	0.098
>40	8 (5.2)	20 (13.1)	28 (9.2)	0.62 (0.19, 2.01)	0.427
Religion					
Orthodox	47 (30.7)	61 (39.9)	108 (35.3)	0.57 (0.34, 0.96)	0.033
Muslim	17 (11.1)	28 (18.3)	45 (14.7)	0.45 (0.22, 0.90)	0.025
Others	16 (10.5)	10 (6.5)	26 (8.5)	1.18 (0.50, 2.81)	0.703
Protestant	73 (47.7)	54 (35.3)	127 (41.5)	1	0.333
Ethnic group					
Nuer	38 (24.8)	22 (14.4)	60 (19.6)	1.60 (0.81, 3.18)	0.179
Oromo	30 (19.6)	28 (18.3)	58 (19.0)	0.99 (0.50, 1.96)	0.984
Amhara	19 (12.4)	25 (16.3)	44 (14.4)	0.70 (0.34, 1.48)	0.355
Others	25 (16.3)	40 (26.1)	65 (21.2)	0.58 (0.30, 1.13)	0.108
Agnuha	41 (26.8)	38 (24.8)	79 (25.8)	1	
Mother's education					
Cannot read and write	48 (31.4)	25 (16.3)	73 (23.9)	2.35 (1.20, 4.63)	0.013
Read and write only	21 (13.7)	30 (19.6)	51 (16.7)	0.86 (0.41, 1.78)	0.682
Primary	28 (18.3)	34 (22.2)	62 (20.3)	1.01 (0.51, 2.01)	0.979
Secondary	25 (16.3)	26 (17.0)	51 (16.7)	1.18 (0.57, 2.43)	0.657
Above secondary	31 (20.3)	38 (24.8)	69 (22.5)	1	
Father's education					
Cannot read and write	19 (12.4)	10 (6.5)	29 (9.5)	1.94 (0.86, 4.38)	0.111
Read and write only	8 (5.2)	14 (9.2)	22 (7.2)	0.58 (0.23, 1.45)	0.247
Primary	11 (7.2)	8 (5.2)	19 (6.2)	1.40 (0.54, 3.64)	0.486
Secondary	18 (11.8)	22 (14.4)	40 (13.1)	0.83 (0.42, 1.65)	0.605
Above secondary	97 (31.7)	99 (64.5)	196 (64.1)	1	
Mother's occupation					
Housewife	99 (64.7)	70 (45.8)	169 (55.2)	2.23 (1.38, 3.62)	0.001
Employee	45 (29.4)	71 (46.4)	116 (37.9)	1	
Merchant	9 (5.9)	12 (7.8)	21 (6.9)	1.18 (0.46, 3.03)	0.726
Father's occupation					
Farmer	21 (13.7)	14 (9.2)	35 (11.4)	1.54 (0.75, 3.18)	0.243
Employee	112 (73.2)	115 (75.1)	227 (74.2)	1	
Merchant	20 (13.1)	24 (15.7)	44 (14.4)	0.86 (0.45, 1.64)	0.637

Child-related factors					
Sex of the child					
Female	69 (45.1)	86 (56.2)	155 (50.7)	0.64 (0.41, 1.01)	0.052
Male	84 (54.9)	67 (43.8)	151 (49.3)	1	
Age of the child (in month)					
1–12	53 (34.6)	37 (24.2)	90 (29.4)	1.63 (0.95, 2.79)	0.074
13–24	35 (22.9)	42 (27.5)	77 (25.2)	0.95 (0.54, 1.66)	0.854
≥24	65 (42.5)	74 (48.4)	139 (45.4)	1	

Household-related factors					
Family size					
≤3	44 (28.8)	48 (31.4)	92 (30.1)	1	
4–6	90 (58.8)	71 (46.4)	161 (52.6)	1.38 (0.83, 2.31)	0.216
>6	19 (12.4)	34 (22.2)	53 (17.3)	0.61 (0.30, 1.22)	0.163
Number of under-five in the household					
1–2	143 (93.5)	141 (92.2)	284 (92.8)	1	
3–4	10 (6.5)	12 (7.8)	22 (7.3)	0.82 (0.34, 1.96)	0.658

Distance and transportation-related factors					
Distance					
>3 km	29 (19.0)	21 (13.7)	50 (16.3)	1.47 (0.80, 2.71)	0.218
≤3 km	124 (81.0)	132 (86.3)	256 (83.7)	1	
Transport systems					
Foot	38 (24.8)	34 (22.2)	72 (23.5)	1.16 (0.68, 1.96)	0.590
Car (taxi)	115 (75.2)	119 (77.8)	234 (76.5)	1	
Family monthly income (in ETB) (*n* = 255)					
≤1300.00	11 (9.2)	17 (12.5)	28 (11.0)	0.71 (0.32, 1.59)	0.408
>1300.00	108 (90.8)	119 (87.5)	227 (89.0)	1	

Total	119 (46.67)	136 (53.33)	255 (100)		

1, reference group.

**Table 2:** Behavior related factors of the caregivers of under-five children at Gambella town, March 2017.

Variables	Delay status in malaria treatment		Total (%)	Crude OR (95% CI)	*p* value
	Case (*N* = 153) number (%)	Control (*N* = 153) number (%)			
Fear of drugs side effect					
Yes	33 (21.6)	40 (26.1)	73 (23.9)	0.78 (0.46, 1.32)	0.348
No	120 (78.4)	113 (73.9)	233 (76.1)	1	

History of child death					
No	138 (90.2)	119 (77.8)	257 (84.0)	2.63 (1.36, 5.06)	0.004
Yes	15 (9.8)	34 (22.2)	49 (16.0)	1	

Distrust health provider					
Yes	18 (11.8)	11 (7.2)	29 (9.5)	1.72 (0.78, 3.78)	0.176
No	135 (88.2)	142 (92.8)	277 (90.5)	1	

Self-medication					
Yes	29 (19.0)	20 (13.1)	49 (16.0)	1.55 (0.84, 2.89)	0.163
No	124 (81.0)	133 (86.9)	257 (84.0)	1	

Visiting traditional healers					
Yes	17 (11.1)	12 (7.8)	29 (9.5)	1.47 (0.68, 3.19)	0.331
No	136 (88.9)	141 (92.2)	277 (90.5)	1	

Alcohol drinking					
Yes	21 (13.7)	20 (13.1)	41 (13.4)	1.06 (0.55, 2.04)	0.867
No	132 (86.3)	133 (86.9)	265 (86.6)	1	

Khat chewing					
Yes	29 (19.0)	11 (7.2)	40 (13.1)	3.02 (1.45, 6.29)	0.003
No	124 (81.0)	142 (92.8)	266 (86.9)	1	

Fear of treatment cost					
Yes	35 (22.9)	34 (22.2)	69 (22.5)	1.038 (0.61, 1.77)	0.891
No	118 (77.1)	119 (77.8)	237 (77.5)	1	

Knowledge					
Poor	28 (18.3)	21 (13.7)	49 (16.0)	1.41 (0.76, 2.61)	0.277
Good	125 (81.7)	132 (86.3)	257 (84.0)	1	

1, reference group.

**Table 3:** Determinants for the delay in seeking malaria treatment for under-five children at Gambella town, March 2017.

Variables	Delay status in malaria treatment		Crude OR (95% CI)	Adjusted OR (95% CI)
	Cases number	Controls number		
Mothers occupation				
Housewife	99	70	2.23 (1.38, 3.62)	2.50 (1.47, 4.22)^*∗*^
Employee	45	71	1	1
Merchant	9	12	1.18 (0.46, 3.03)	1.84 (0.66, 5.16)

History of child death				
Yes	15	34	1	1
No	138	119	2.63 (1.36, 5.06)	3.70 (1.79, 7.64)^*∗*^

Khat chewing				
Yes	29	11	3.02 (1.45, 6.29)	3.50 (1.57, 7.68)^*∗*^
No	124	142	1	1

Ethnic group				
Agnuha	41	38	1	1
Nuer	38	22	1.60 (0.81, 3.18)	1.70 (0.81, 3.55)
Oromo	30	28	0.99 (0.50, 1.96)	1.06 (0.52, 2.19)
Amhara	19	25	0.70 (0.34, 1.48)	0.53 (0.25, 1.09)
Others	25	40	0.58 (0.30, 1.13)	0.56 (0.25, 1.26)

1, reference group. ^*∗*^*p* value <0.05 in multivariable analysis.

## Data Availability

The datasets used and/or analyzed during the current study are available from the corresponding author upon reasonable request.

## Abbreviations

AOR: Adjusted odds ratio
COR: Crude odds ratio
ETB: Ethiopian birr
GC: Gregorian calendar
NSP: National strategic plan
OR: Odds ratio
SD: Standard deviations
SPSS: Statistical Package for Social Science
WHO: World Health Organization.
